# Supplementary figures and images for: Fluorescent Protein Inserts in between NC and SP2 Are Tolerated for Assembly, Release and Maturation of HIV with Limited Infectivity
Source: Viruses. 2019 Oct 23;11(11):973. doi: 10.3390/v11110973 (PMC6893430; doi:10.3390/v11110973)

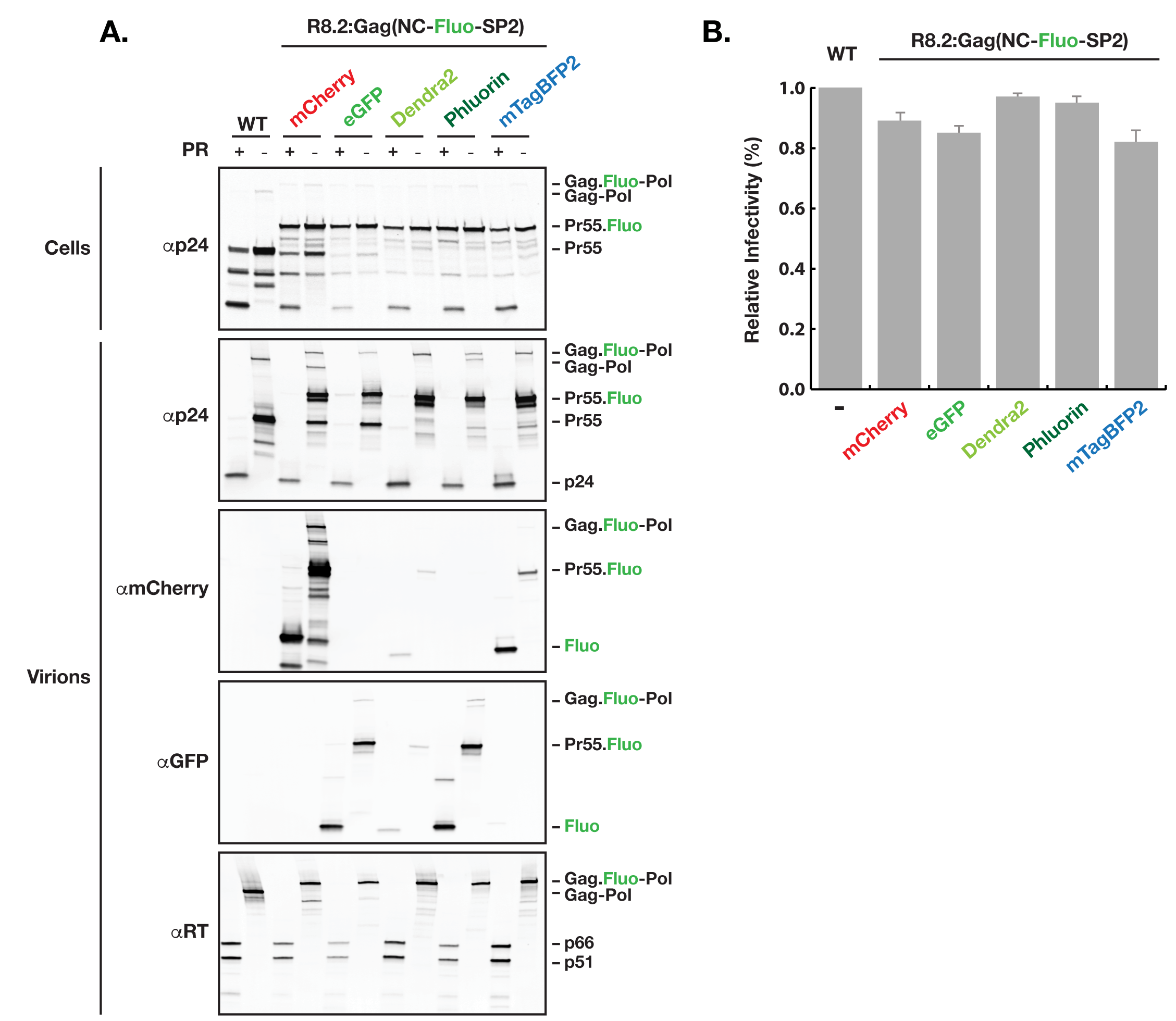

Supplement: Supplementary file 1 [file viruses-11-00973-s001.zip › Fig_S1.tif]
